# Identification of small‐molecule elastase inhibitors as antagonists of IL‐36 cytokine activation

**DOI:** 10.1002/2211-5463.12406

**Published:** 2018-03-25

**Authors:** Graeme P. Sullivan, Pavel B. Davidovich, Sylvia Sura‐Trueba, Ekaterina Belotcerkovskaya, Conor M. Henry, Danielle M. Clancy, Anna Zinoveva, Tazhir Mametnabiev, Alexander V. Garabadzhiu, Seamus J. Martin

**Affiliations:** ^1^ Molecular Cell Biology Laboratory Department of Genetics The Smurfit Institute Trinity College Dublin 2 Ireland; ^2^ Cellular Biotechnology Laboratory Saint‐Petersburg State Institute of Technology Russia

**Keywords:** elastase, IL‐1 family, IL‐36, inflammation, protease, psoriasis

## Abstract

IL‐1 family cytokines act as apical initiators of inflammation in many settings and can promote the production of a battery of inflammatory cytokines, chemokines and other inflammatory mediators in diverse cell types. IL‐36α, IL‐36β and IL‐36γ, which belong to the extended IL‐1 family, have been implicated as key initiators of skin inflammation in psoriasis. IL‐36γ is highly upregulated in lesional skin from psoriatic individuals, and heritable mutations in the natural IL‐36 receptor antagonist result in a severe form of psoriasis. IL‐36 family cytokines are initially expressed as inactive precursors that require proteolytic processing for activation. The neutrophil granule‐derived protease elastase proteolytically processes and activates IL‐36α and IL‐36γ, increasing their biological activity ~ 500‐fold, and also robustly activates IL‐1α and IL‐33 through limited proteolytic processing. Consequently, inhibitors of elastase activity may have potential as anti‐inflammatory agents through antagonizing the activation of multiple IL‐1 family cytokines. Using *in silico* screening approaches, we have identified small‐molecule inhibitors of elastase that can antagonize activation of IL‐36γ by the latter protease. The compounds reported herein may have utility as lead compounds for the development of inhibitors of elastase‐mediated activation of IL‐36 and other IL‐1 family cytokines in inflammatory conditions, such as psoriasis.

**Abbreviations**
AAPV‐AMC: methoxy‐succinyl‐Ala‐Ala‐Pro‐Val‐7‐amino‐4‐trifluoromethylcoumarin
ABS: absorbance
AZD9668: Alvelestat
CatG: cathepsin G
CGi: cathepsin G inhibitor
CTRL: control
CXCL1: chemokine (C‐X‐C motif) ligand 1
DHPI: dihydropyrimidine
FLF‐sBzl: succinyl‐Phe‐Leu‐Phe‐thiobenzyl ester
H&E: haematoxylin and eosin
HNE: human neutrophil elastase
IL: interleukin
IL‐36RA: interleukin‐36 receptor antagonist
IL‐36R: interleukin‐36 receptor
NE: neutrophil elastase
NEi: neutrophil elastase inhibitor
PMA: phorbol 12‐myristate 13‐acetate
PR3: proteinase‐3
PRB: protease reaction buffer
Rec: recombinant
RFU: relative fluorescence unit
RPMI: Roswell Park Memorial Institute
SCID: severe combined immune‐deficient
SN: supernatant
TNF: tumour necrosis factor
UT: untreated
VS: virtual screening
WEHD‐AMC: Ac‐Trp‐Glu‐His‐Asp‐7‐amino‐4‐trifluoromethylcoumarin
z‐VAD‐fmk: carbobenzoxy‐valyl‐alanyl‐aspartyl‐[O‐methyl]‐fluoromethylketone

IL‐1 family cytokines, which include the recently described IL‐36α, IL‐36β and IL‐36γ proteins, are among the first cytokines produced in response to infection or injury and play key roles in the initiation of inflammation as a consequence 1, 2, 3, 4. IL‐1 family cytokines can initiate the production of many additional cytokines from diverse cell types, such as tissue macrophages and dendritic cells, as well as keratinocytes and endothelial cells lining local blood vessels 5, 6, 7, 8, 9, 10, 11. IL‐36α, IL‐36β and IL‐36γ are encoded by distinct genes, but all signal via the same receptor, and much evidence now suggests that one or more of these cytokines play an important role in psoriasis 12, 13, 14, 15, 16, 17, 18, 19, 20, 21.

Individuals that carry loss‐of‐function mutations in the IL‐36 receptor antagonist (IL‐36RA) display a severe form of psoriasis, called generalized pustular psoriasis 16, 17, 18, 19, 20. Furthermore, expression of all three IL‐36 family members has been found to be dramatically elevated (100‐fold) in skin biopsies from individuals with the most common form of psoriasis, psoriasis vulgaris, compared with nonlesional skin from the same individuals, or nonaffected controls 13, 14. This suggests that deregulated IL‐36 cytokine signalling is sufficient to initiate psoriatic‐type skin inflammation. Moreover, deregulated expression of IL‐36α in the mouse leads to a psoriasis‐like condition that is exacerbated with the skin irritant, phorbol acetate 12. In addition, transplantation of human psoriatic lesions onto immunodeficient (SCID) mice produces a psoriasis‐like condition that is ameliorated through IL‐36 receptor neutralization 13.

As is common within the extended IL‐1 cytokine family, IL‐36α, IL‐36β and IL‐36γ are all expressed as leaderless cytokines that lack biological activity unless cleaved within their N termini 11, 22, 23. Thus, limited proteolytic processing of IL‐36 family cytokines to remove up to 15 N‐terminal amino acids dramatically increases their pro‐inflammatory activity by over 500‐fold 11, 22, 24, 25. We have recently reported that the neutrophil‐derived proteases cathepsin G (CatG) and elastase are potent IL‐36‐processing proteases, with elastase playing a major role in the processing and maturation of IL‐36α and IL‐36γ 11, 25. Furthermore, IL‐1α and IL‐33 are also proteolytically processed and activated by elastase [reviewed in Ref. 26]. Because deregulated IL‐36 cytokine activation appears to be instrumental in the initiation of inflammation in the skin barrier, inhibitors of IL‐36 activation and/or downstream activity are likely to have potential for the treatment of inflammatory skin conditions. Neutrophil infiltrates are common in psoriasis, and these cells are the major source of elastase 27, 28, 29. Thus, small‐molecule inhibitors of neutrophil elastase (NE) may have significant potential as inhibitors of IL‐36 activation in psoriasis.

Here, we have used *in silico* screening approaches to identify small‐molecule inhibitors of elastase, followed by functional testing of candidates. We show that these inhibitors are capable of antagonizing elastase‐mediated processing and activation of IL‐36γ, suggesting that these compounds may be useful leads for the generation of therapeutic modulators of IL‐36 cytokine activity in inflammatory conditions.

## Results

### Elastase processes and activates IL‐36γ

**Figure 1:**
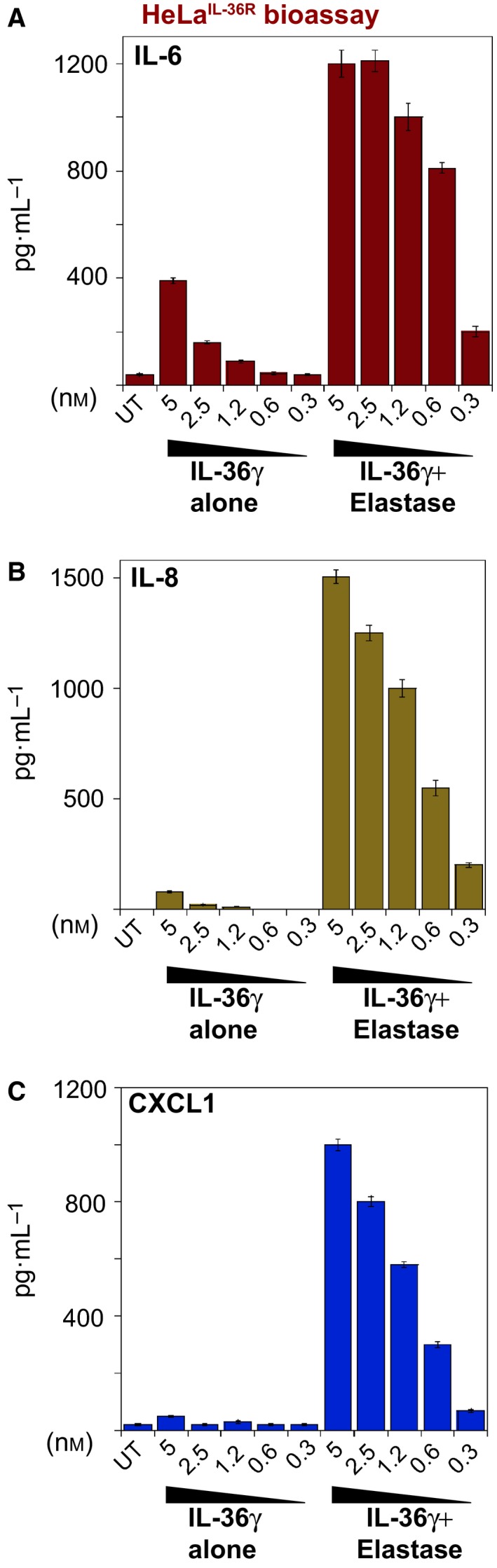
IL‐36γ is processed and activated by NE. HeLa^IL‐36R^ cells were either left untreated or were treated with the indicated concentrations of full‐length recombinant human IL‐36γ (ranging from 5 to 0.3 nm), or the same amounts of IL‐36γ that had been pre‐incubated for 2 h at 37 °C with purified HNE (50 nm). Twenty‐four hours after incubation with either full‐length or elastase‐processed IL‐36γ preparations, cytokine concentrations in the culture SNs were determined by ELISA. The following cytokines were measured: (A) IL‐6, (B) IL‐8 and (C) CXCL1. Results shown are representative of at least three independent experiments. Error bars represent the mean ± SEM of triplicate determinations from a representative experiment.

Similar to other members of the extended IL‐1 family, such as IL‐1β and IL‐18 26, IL‐36 cytokines possess minimal pro‐inflammatory activity as full‐length proteins and require N‐terminal processing for activation 11, 22. However, as we have recently reported 11, 25, IL‐36γ is robustly activated upon incubation with elastase (Fig. 1), a protease that is released in large quantities into the extracellular space from the cytoplasmic granules of activated neutrophils. Elastase processes IL‐36γ at Val15, liberating a new N terminus that unleashes the pro‐inflammatory activity of this cytokine, possibly through provoking a conformational change in the latter or through eliminating steric interference within the receptor‐binding domain of this cytokine 11. Of note, previous studies have shown that IL‐36γ is dramatically upregulated at the mRNA and protein levels in lesional skin from psoriasis patients, compared with unaffected skin from the same individuals, or from control subjects 13, 14, 24, 30. Coupled with observations that loss‐of‐function mutations in the natural IL‐36 receptor antagonist promote a highly severe form of psoriasis 16, 17, 18, 19, 20, this suggests that IL‐36γ may be an important driver of the inflammation seen in this condition.

Migration of circulating neutrophils into peripheral tissues is a major amplifier of inflammation and is commonly seen in psoriatic lesions. Neutrophil‐derived proteases such as elastase and CatG, although generally thought of as antimicrobial enzymes, are also potent instigators of inflammation 4, 11, 25, 26, most likely through processing and activation of IL‐1 family cytokines such as IL‐36α and IL‐36γ. Therefore, inhibitors of NE may have therapeutic potential as anti‐inflammatory agents through antagonizing processing and activation of multiple IL‐1 family cytokines.

### Identification of candidate elastase inhibitors using an *in silico* screening approach

**Figure 2:**
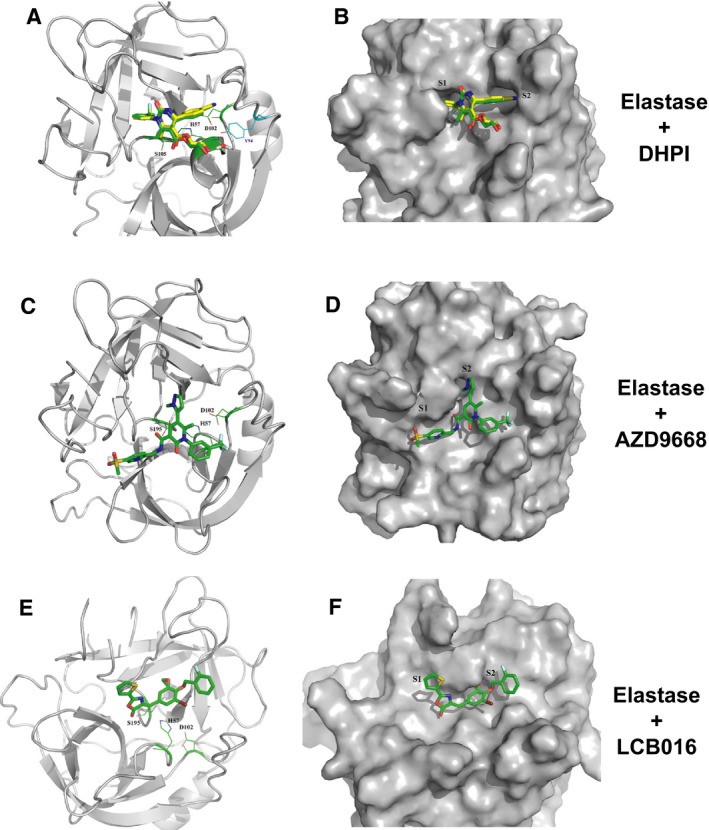
Structure‐based *in silico* screening strategy to identify candidate elastase inhibitors. (A, B) *In silico* docking of the elastase crystal structure with the elastase inhibitor DHPI. (C, D) *In silico* docking of the elastase crystal structure with the elastase inhibitor Alvelestat/AZD9668. (E, F) Representation of the binding pose of newly identified LCB016 inhibitor and interaction interface of LCB016 in the active site of NE. Magenta arrow represents H‐bond, and green represents π–π contacts.

To identify novel small‐molecule inhibitors of elastase, we performed *in silico* screening of the elastase crystal structure with an in‐house compound library comprised of over 100 000 unique molecular entities (Saint Petersburg Technical University). Using molecular dynamics simulation, compounds were docked in multiple poses into the substrate‐binding pocket of elastase, as illustrated by the examples presented in Fig. 2. Using this approach, and guided by known inhibitors of elastase such as dihydropyrimidine (DHPI) (Fig. 2A,B) and Alvelestat/AZD9668 (Fig. 2C,D), we identified a small molecule, designated LCB016, which fit the binding parameters expected of a candidate inhibitor (Fig. 2E,F).

### LCB016 and derivatives thereof exhibit elastase‐inhibitory activity

**Figure 3:**
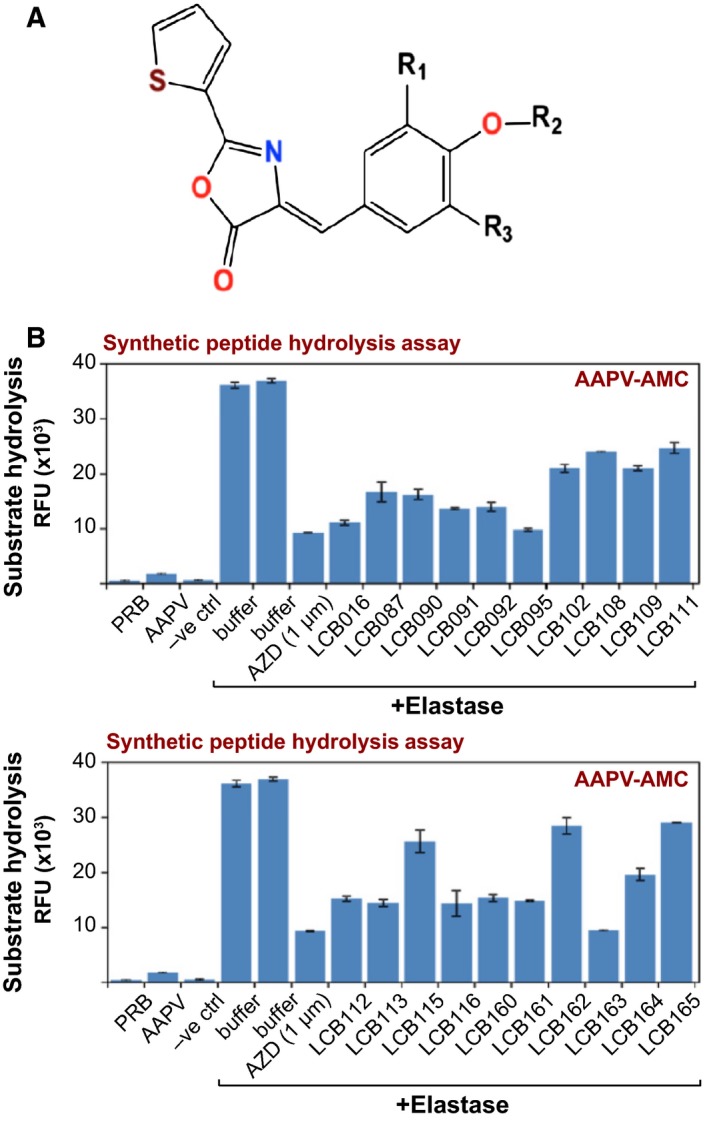
Several candidates identified by *in silico* screening exhibit inhibitory activity towards purified elastase. (A) General structural formula of LCB016 NE azolactone inhibitor. A series of azolactone analogues were synthesized with substitutions at position R_1_, R_2_ or R_3_. (B) LCB016 and its derivatives (10 μm) were incubated with NE for 2 h at 37 °C. Elastase enzymatic activity was measured by hydrolysis of the elastase substrate peptide Suc‐AAPV‐AMC (50 μm). AZD9668 (1 μm) was used as a positive control for elastase inhibition.

LCB016 has an azolactone structure (Fig. 3A); therefore, a series of azolactone analogues were synthesized (LCB001–LCB165; Table 1), which were then assessed for their ability to antagonize elastase activity, initially using a synthetic substrate (AAPV‐AMC) hydrolysis assay. As Fig. 3B demonstrates, LCB016 and several of its derivatives were found to inhibit NE activity as assessed by the ability of these compounds to antagonize hydrolysis of the synthetic elastase substrate peptide Suc‐AAPV‐AMC. Substitution of the bromine in LCB016 by hydrogen (LCB108, LCB109, LCB111) led to a significant decrease in calculated binding energy to elastase (data not shown), and consequently to diminished inhibitor potency (Fig. 3B). Substitution of the ethoxy group to smaller methoxy in LCB016 also led to a decrease in binding energy (Table 1 and Fig. 3B). Furthermore, the presence of the benzoxy group was found to be essential to the binding (data not shown).

**Table 1:** List of azolactone NE inhibitors and derivatives

ID number	Substituents
LCB016	R_1_ = Br, R_2_ = 2‐fluoro‐Bn, R_3_ = OEt
LCB087	R_1_ = Br, R_2_ = 2‐chloro‐Bn, R_3_ = OMe
LCB090	R_1_ = Cl, R_2_ = 2‐chloro‐Bn, R_3_ = OMe
LCB091	R_1_ = Br, R_2_ = 2‐bromo‐Bn, R_3_ = OMe
LCB092	R_1_ = Cl, R_2_ = 2‐fluoro‐Bn, R_3_ = OEt
LCB095	R_1_ = I, R_2_ = 4‐iodo‐Bn, R_3_ = OEt
LCB102	R_1_ = Br, R_2_ = Br, R_3_ = Br
LCB108	R_1_ = H, R_2_ = Et, R_3_ = H
LCB109	R_1_ = H, R_2_ = Bn, R_3_ = H
LCB111	R_1_ = H, R_2_ = 2‐fluoro‐Bn, R_3_ = OEt
LCB112	R_1_ = Br, R_2_ = 4‐fluoro‐Bn, R_3_ = OEt
LCB113	R_1_ = Br, R_2_ = 2‐fluoro‐Bn, R_3_ = OMe
LCB115	R_1_ = Br, R_2_ = 3‐fluoro‐Bn, R_3_ = OEt
LCB116	R_1_ = Br, R_2_ = 2‐fluoro‐Bn, R_3_ = OEt
LCB160	R_1_ = Cl, R_2_ = 2‐fluoro‐Bn, R_3_ = OEt
LCB161	R_1_ = I, R_2_ = 2‐fluoro‐Bn, R_3_ = OEt
LCB162	R_1_ = NO_2_, R_2_ = 2‐fluoro‐Bn, R_3_ = OEt
LCB163	Thiophene is substituted on bioisosteric furan ring
LCB164	Thiophene is substituted on benzene ring
LCB165	Azolactone ring is substituted with sulfur

**Figure 4:**
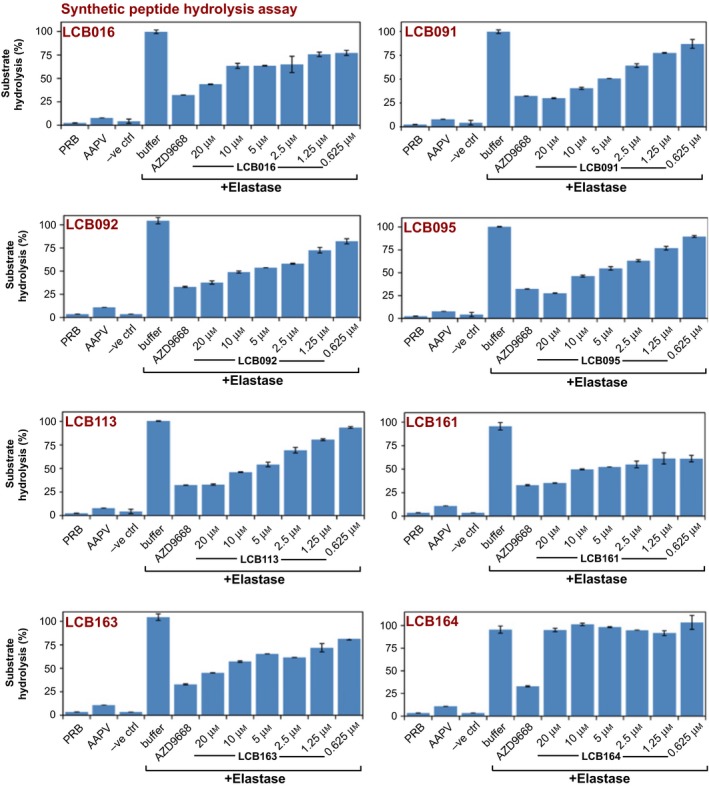
Azolactone derivatives inhibit elastase activity. LCB016 and its derivatives were titrated against purified NE. Azolactone derivatives were incubated with elastase for 2 h at 37 °C, as indicated. AZD9668 (1 μm) was used as a positive control for elastase inhibition. Elastase enzymatic activity was measured by hydrolysis of the elastase substrate peptide Suc‐AAPV‐AMC (50 μm).

**Figure 5:**
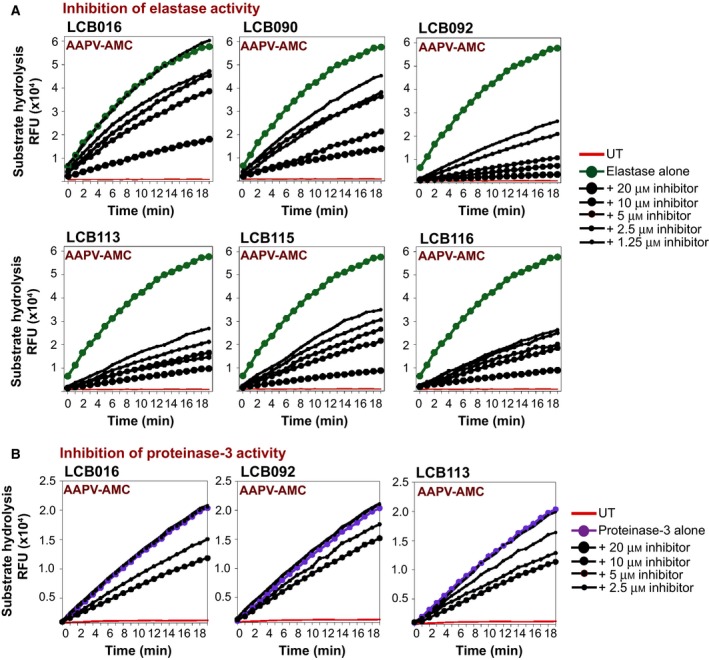
Kinetic analysis of elastase inhibition and specificity of azolactone derivatives. (A) Hydrolysis of the synthetic peptide substrate AAPV‐AMC by a fixed concentration of purified elastase (50 nm), in the presence or absence of the indicated concentrations of the candidate elastase inhibitors. (B) Hydrolysis of the synthetic peptide substrate AAPV‐AMC by a fixed concentration of purified PR3 (200 nm), in the presence or absence of the indicated concentrations of the candidate elastase inhibitors.

Based on these preliminary screening results, we performed titrations of LCB016 and several of its derivatives (LCB091, LCB092, LCB095, LCB113, LCB161, LCB163 and LCB164) against purified elastase (Fig. 4). Once again, we observed reproducible elastase‐inhibitory activity of LCB016 and several of its derivatives, with LCB092 and LCB113 exhibiting greater potency than LCB016 (Fig. 4). We also performed time‐course analyses of LCB016 and the most promising derivatives over a range of drug concentrations, and as Fig. 5A illustrates, LCB092, LCB113 and LCB116 exhibited the greatest potency towards elastase.

### Specificity of candidate elastase inhibitors towards other inflammatory proteases

**Figure 6:**
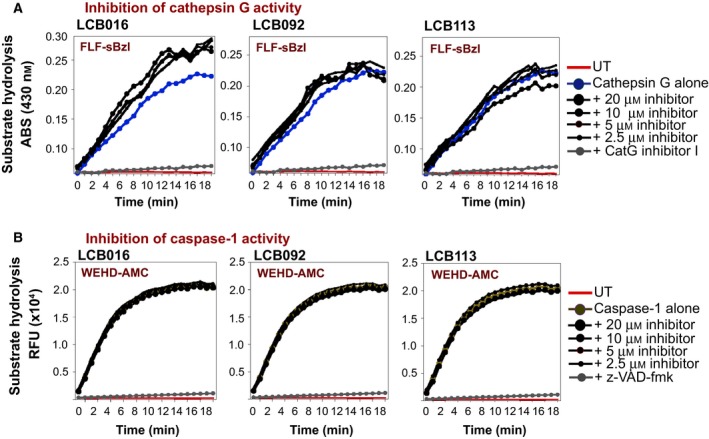
Azolactone derivatives do not inhibit neutrophil CatG or caspase‐1. (A) Hydrolysis of the synthetic CatG substrate FLF‐sBzl by a fixed concentration of purified CatG (50 nm), in the presence or absence of the indicated concentrations of the candidate elastase inhibitors. Cathepsin G inhibitor I (CatG inhibitor I, 5 μm) served as a positive control. (B) Hydrolysis of the synthetic caspase‐1 substrate WEHD‐AMC by a fixed concentration of recombinant human caspase‐1 (1 : 100), in the presence or absence of the indicated concentrations of the candidate elastase inhibitors. z‐VAD‐fmk (5 μm) served as a positive control.

There is approximately 56% structural similarity between NE and proteinase‐3 (PR3), and these proteases exhibit almost identical substrate preferences 11. Thus, LCB016 and its derivatives also exhibited some inhibitory activity towards PR3, as expected (Fig. 5B). However, no cross‐inhibitory effect was observed towards the other major neutrophil protease, CatG (Fig. 6A). Furthermore, caspase‐1, which plays a key inflammatory role through processing and activation of IL‐1β and IL‐18, was also unaffected by the candidate elastase inhibitors (Fig. 6B). Collectively, these data suggest that the compounds identified herein exhibit good specificity towards NE.

### Small‐molecule elastase inhibitors can antagonize IL‐36γ activation and cytokine production downstream

**Figure 7:**
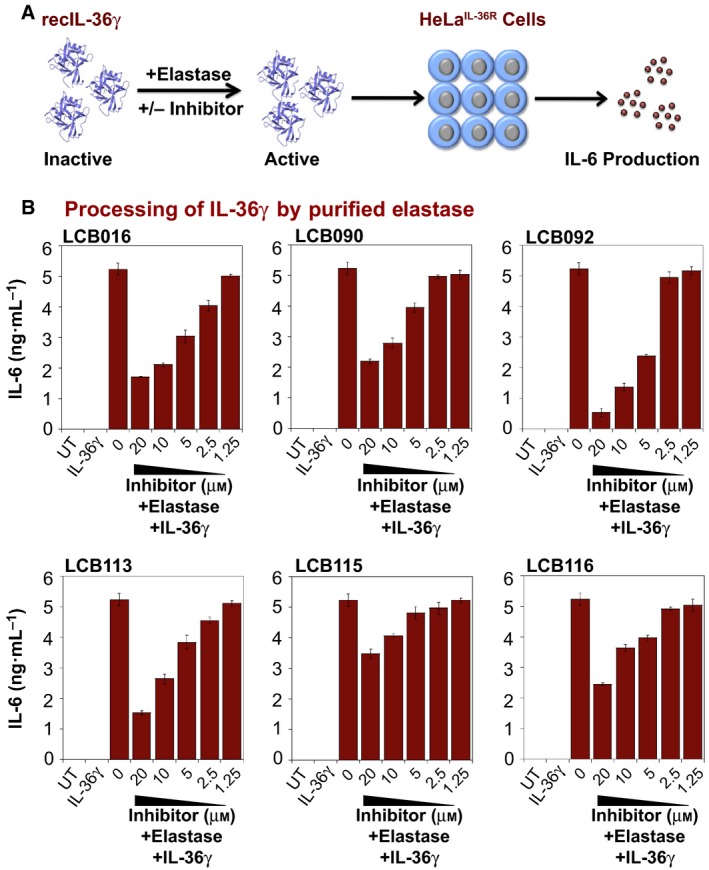
Inhibition of elastase‐mediated IL‐36γ activation by LCB016 and derivatives. (A) Schematic overview of the IL‐36 activity bioassay. (B) Purified recombinant IL‐36γ was incubated for 2 h at 37 °C in the presence or absence of elastase (50 nm), either alone or in combination with the indicated small‐molecule elastase inhibitors. Reaction products were then added to HeLa^IL‐36R^ cells, such that the final concentration of IL‐36γ was 500 pm. After 24 h, IL‐6 cytokine concentrations in cell culture SNs were determined by ELISA. Results shown are representative of at least three independent experiments. Error bars represent the mean ± SEM of triplicate determinations from a representative experiment.

Based on the synthetic substrate hydrolysis data, six compounds (LCB016, LCB090, LCB092, LCB113, LCB115 and LCB116) were selected for further testing in the more physiologically relevant context of elastase‐mediated IL‐36γ processing. Here, we incubated recombinant full‐length IL‐36γ in the presence or absence of elastase, either alone or in combination with the candidate elastase inhibitors (Fig. 7A). The products of the latter reactions were then incubated with HeLa^IL‐36R^ cells, which respond to active (i.e. elastase‐processed) IL‐36γ by secreting a range of pro‐inflammatory cytokines and chemokines such as IL‐6, IL‐8 and CXCL1 (Fig. 7A) 11, 23. As Fig. 7B illustrates, while LCB016 and some of its derivatives clearly suppressed elastase‐mediated activation of IL‐36γ, as indicated by the inhibition of IL‐6 synthesis from HeLa^IL‐36R^ cells, LCB092 and LCB113 exhibited greater potency in this regard, in agreement with the synthetic substrate hydrolysis assays (Fig. 5A).

**Figure 8:**
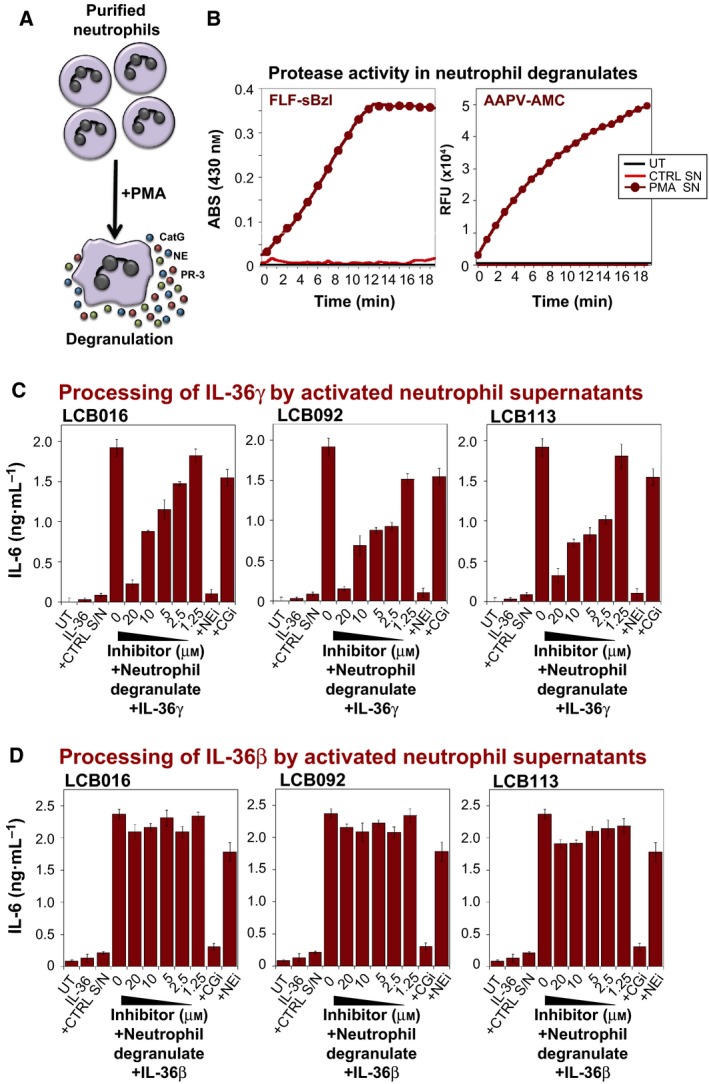
Novel elastase inhibitors suppress IL‐36γ activation by proteases released from human neutrophil degranulate SNs. (A) Schematic representation of PMA‐induced neutrophil degranulation. (B) Assessment of elastase and CatG activity in SNs from untreated versus PMA‐activated human neutrophils. Protease activity was assessed through hydrolysis of FLF‐sBzl (CatG activity) or AAPV‐AMC (elastase/PR3 activity), as before. (C) Purified recombinant human IL‐36γ was incubated for 2 h at 37 °C in the presence or absence of control (unstimulated) or PMA‐activated neutrophil SNs (1 : 8), either alone or in combination with the indicated small‐molecule elastase inhibitors. Neutrophil cathepsin G inhibitor I (CGi) and elastase inhibitor IV (NEi) served as controls. Reaction products were then added to HeLa^IL‐36R^ cells, such that the final concentration of IL‐36γ was 500 pm. After 24 h, IL‐6 cytokine concentrations in cell culture SNs were determined by ELISA. (D) Purified recombinant IL‐36β was incubated for 2 h at 37 °C in the presence or absence of control (unstimulated) or PMA‐activated neutrophil SNs (1 : 8), either alone or in combination with the indicated small‐molecule elastase inhibitors. Neutrophil cathepsin G inhibitor I (CGi) and elastase inhibitor IV (NEi) served as controls. Reaction products were then added to HeLa^IL‐36R^ cells, such that the final concentration of IL‐36β was 500 pm. After 24 h, IL‐6 cytokine concentrations in cell culture SNs were determined by ELISA. Results shown are representative of at least three independent experiments. Error bars represent the mean ± SEM of triplicate determinations from a representative experiment.

To explore whether the inhibitors identified herein could also inhibit IL‐36γ processing and activation by elastase released from activated neutrophils, we activated purified human neutrophils via treatment with phorbol 12‐myristate 13‐acetate (PMA; Fig. 8A). The latter treatment triggered the release of multiple neutrophil granule proteases, including elastase, PR3 and CatG, into the extracellular space as expected (Fig. 8B). Using activated neutrophil supernatants (SNs) as a source of elastase to process and activate IL‐36γ, we observed that the lead compounds (LCB016, LCB092 and LCB113) robustly suppressed activation of the latter by neutrophil‐derived proteases (Fig. 8C). As we have reported previously 11, IL‐36β is predominantly activated by CatG, and in accordance with this, we found that our elastase‐inhibitory compounds had minimal effects on IL‐36β activation by neutrophil degranulate preparations. However, inhibition of CatG activity, using a commercial inhibitor of the latter, robustly suppressed activation of IL‐36β by activated neutrophil degranulate preparations (Fig. 8D).

In summary, we have identified a number of compounds that are capable of acting as inhibitors of elastase and may have utility as lead compounds designed to suppress inflammation through blocking the actions of this protease against cytokines such as IL‐36γ.

## Discussion

Here, we report the identification of several small molecules that exhibit significant inhibitory activity against NE and can antagonize the processing and activation of IL‐36γ by the latter. Because elastase has also been implicated in the processing and activation of IL‐1α, IL‐1β, IL‐33 and other cytokines 26, the compounds reported herein are also likely to suppress the activation of multiple members of the extended IL‐1 family. Further experiments will explore the latter possibility and will also explore the activity of these compounds in animal models of psoriasis and other inflammatory diseases.

Neutrophil infiltration is a hallmark of a number of skin‐related inflammatory diseases. In particular, psoriatic plaques are heavily infiltrated with neutrophils, dendritic cells, macrophages and T cells 27, 28, 31. Neutrophils are well‐known first responder cells of the innate immune system and play a central role in the early stages of infection or tissue damage 32, 33, 34. Although it is widely appreciated that release of neutrophil proteases (through degranulation or neutrophil extracellular trap [NET] formation) can exert profound antimicrobial effects during infection, the latter proteases can also cause extensive tissue damage and exacerbate inflammation 35, 36, 37. Psoriasis is often preceded by tissue damage or abrasion, called the Koebner reaction 38, which is most likely due to liberation of cytokines such as IL‐36 and other IL‐1 family cytokines into the extracellular space due to necrosis. Upon release into the interstitial fluid, the latter cytokines can then encounter and become processed by proteases, such as elastase, liberated from activated neutrophils. Thus, damage to keratinocytes resulting in the liberation of IL‐36 cytokines, either as a result of microbial infection or as a consequence of tissue trauma, may play an important initiating role in psoriasis, especially in individuals lacking endogenous buffers of IL‐36 activity, such as deficiency in the IL‐36R antagonist 16, 17, 18, 19, 20. Because previous studies have also implicated NE in the activation of IL‐1α 39, IL‐1β 40, IL‐18 41 and IL‐33 42 and IL‐36 receptor antagonist 43, our observations also suggest that this protease may serve as a therapeutic target for suppressing the activation of multiple IL‐1 family cytokines in inflammatory diseases.

Psoriasis is associated with a pro‐inflammatory signature with elevated levels of tumour necrosis factor (TNF), IL‐17C and IL‐36 among other cytokines. At present, multiple biotherapeutics, directed against cytokines such as TNF, IL‐17, IL‐12/IL‐23 and IL‐36, have been approved or are in development for the treatment of psoriasis, as well as other inflammatory conditions. However, although these cytokine‐neutralizing approaches are highly effective, they also suffer from several drawbacks including high cost, a necessity for systemic delivery (which can increase vulnerability to opportunistic infections) and their single‐molecule specificity. The need for systemic delivery of cytokine‐neutralizing biotherapeutics is particularly problematic due to the neutralization of cytokine throughout the body and not just where it is overproduced. A more desirable approach would be to neutralize cytokine only in the affected lesions. Thus, small molecules, such as the compounds reported herein, have the advantage that they are less costly to produce, can be applied directly to affected areas of skin and may simultaneously suppress activation of multiple cytokines that are processed and activated by elastase 11, 29.

## Experimental procedures

### Materials

Suc(OMe)‐AAPV‐AMC was purchased from PeptaNova (Sandhausen, Germany); elastase inhibitor Alvelestat/AZD9668 was purchased from MedChem Express (Solentuna, Sweden). Purified neutrophil‐derived elastase was purchased from Serva (Heidelberg, Germany). Purified neutrophil PR3 and neutrophil CatG were purchased from Calbiochem (Merck), Cork, Ireland. Suc‐FLF‐SBzl, Ac‐WEHD‐AMC and z‐VAD‐fmk were purchased from Bachem (Bubendorf, Switzerland). Chemical inhibitors of CatG (cathepsin G inhibitor I) and of elastase (elastase inhibitor IV) were purchased from Calbiochem. Unless otherwise indicated, all other reagents were purchased from Sigma Aldrich (Ireland) (Wicklow, Ireland).

### Cell culture

HeLa cells were cultured in RPMI media (Gibco (BioSciences), Dublin, Ireland), supplemented with 5% FBS. HeLa.IL‐36R cell lines were generated by transfection with pCXN2.empty or pCXN2.IL‐1Rrp2 (IL‐36R) plasmids followed by selection using G‐418 antibiotic (Sigma). IL‐36R‐overexpressing clones were expanded from a single cell. Clones were selected by demonstration of acquired optimal responsiveness to active forms of IL‐36 via ELISA. All cells were cultured at 37 °C in a humidified atmosphere with 5% CO_2_.

### Expression and purification of recombinant proteins

Full‐length IL‐36β and IL‐36γ were generated by cloning the human coding sequence in frame with the polyhistidine tag sequence in the bacterial expression vector pET45b. Protein was expressed by addition of 600 μm IPTG to exponentially growing cultures of BL21 strain *Escherichia coli* followed by incubation for 3 h at 37 °C. Bacteria were lysed by sonication and polyhistidine‐tagged proteins were captured using nickel/NTA agarose (Qiagen (UK), Manchester, UK), followed by elution into PBS, pH 7.2, in the presence of 100 mm imidazole. Recombinant polyhistidine‐tagged caspase‐1 and caspase‐3 were expressed and purified as described previously 44.

### *In silico* screening and molecular modelling

Ligand 3D structures were prepared with LigPrep suite in OPLS_2005 force field. The Schrodinger package was used for molecular modelling studies. Glide was used for SM virtual screening (VS) and docking studies; the ligands from the in‐house SM library were treated as flexible. The standard precision protocol was used for VS. The positions of the identified azolactone hit compounds were specified within extra‐precision protocol. Azolactone derivatives were redocked as Z‐isomers corresponding XRD data. The X‐ray human neutrophil elastase (HNE) structure (PDB ID http://www.rcsb.org/pdb/search/structidSearch.do?structureId=1HNE) was used for *in silico* screening; the water and cocrystallized molecules were removed from the protein model. Hydrogens were added using PROPKA program pH 7.4 and their positions optimized. The 25 × 25 × 25 Å^3^ box (with 12 Å diameter ligand mid‐point), centred on the coordinates of ^57^His residue (active site), was used for grid map calculations; the ^94^Tyr, ^54^Ser, ^195^Ser and ^214^Ser hydroxyl groups were allowed to rotate during docking runs. For the validation of the docking protocol, the DHPI ligand was blindly docked to the HNE structure (PDB ID http://www.rcsb.org/pdb/search/structidSearch.do?structureId=3Q77) and the poses of docked ligand and cocrystallized with HNE were aligned (Fig. 2) showing good theory‐to‐experiment correspondence.

### Protease activity assays

Reactions (50 μL, final volume) were carried out in protease reaction buffer (PRB) (50 mm HEPES [pH 7.2], 75 mm NaCl and 0.1% 3‐[(‐cholamidopropyl)dimethylammonio]‐1‐propanesulfonate [CHAPS] [2 mm DTT added only for caspases]) containing Ac‐WEHD‐AMC and Suc(OMe)‐AAPV‐AMC (50 μm, final concentration). Samples were measured using an automated fluorimeter (SPARK 10M; TECAN (UK), Reading, UK) at wavelengths of 430 nm (excitation) and 535 nm (emission). For the Suc‐FLF‐SBzl hydrolysis assay, the substrate was diluted to a final concentration of 300 μm in PRB (50 mm HEPES [pH 7.2], 75 mm NaCl, 0.1% CHAPS and 300 μm 5,5‐dithiobis(2‐nitrobenzoic acid [DTNB]). Cathepsin G hydrolyses the synthetic substrate Suc‐FLF‐sBzl with the release of the thiobenzyl group. The free thiobenzyl group reacts with DTNB and produces a chromophore (3,30,5,50‐tetramethylbenzidine [TNB]) that absorbs at 430 nm. Samples were measured by automated fluorimeter (SPARK 10M TECAN (UK)).

### Protease cleavage assays

Reactions (40–100 μL, final volume) were carried out in PRB (50 mm HEPES [pH 7.2], 75 mm NaCl and 0.1% CHAPS) for 2 h at 37 °C. For IL‐36 bioassays, IL‐36 cytokines were typically cleaved at a 50 nm concentration and subsequently diluted onto target cells at a final concentration ranging from 0.25 to 1 nm.

### Purification of primary neutrophils and preparation of degranulates

Primary human neutrophils were purified from donor whole blood using the Ficoll‐Hypaque gradient method as described previously 11. The purity of the cell preparations (> 90%) was determined by H&E staining of cytospins. To prepare degranulates, neutrophils (10^7^ per treatment) were stimulated in the presence or absence of 50 nm PMA in Hanks' balanced salt solution/0.25% BSA for 1–3 h at 37 °C in a humidified atmosphere with 5% CO_2_. SNs were harvested and clarified by centrifugation at 4 °C (10 000 × ***g*** for 5 min). Neutrophil degranulate aliquots were stored at −80 °C. Experiments involving human samples were carried out in accordance with the regulations of the EU and the Irish Department of Health, and all procedures performed were approved by the Trinity College Dublin Research Ethics Committee (Ethical Approval Number 311217).

### Measurement of cytokines and chemokines

Cytokines and chemokines were measured from cell culture SNs using specific ELISA kits obtained from R&D Systems (human IL‐6, IL‐8, CXCL1). All cytokine assays were carried out using triplicate samples from each culture.

## Author contributions

GPS, PBD, SS‐T and EB performed experiments and analysed data; PBD performed the *in silico* screening; CMH, DMC and AZ generated reagents; TM and AVG provided advice, supervision and comments on data; and SJM conceived the project, supervised the study and wrote the manuscript.
